# DEFLATE Compression Algorithm Corrects for Overestimation of Phylogenetic Diversity by Grantham Approach to Single-Nucleotide Polymorphism Classification

**DOI:** 10.3390/ijms15058491

**Published:** 2014-05-13

**Authors:** Arran Schlosberg, Brian Y. H. Lam, Giles S. H. Yeo, Roderick J. Clifton-Bligh

**Affiliations:** 1 Kolling Institute of Medical Research, Royal North Shore Hospital, Pacific Hwy, St Leonards, NSW 2065, Australia; E-Mail: jclifton@med.usyd.edu.au; 2 Sydney Medical School, the University of Sydney, NSW 2006, Australia; 3 University of Cambridge Metabolic Research Laboratories, Box 289, Level 4 Wellcome Trust-MRC Institute of Metabolic Science, Addenbrooke’s Hospital, Hills Road, Cambridge CB2 0QQ, UK; E-Mails: yhbl2@cam.ac.uk (B.Y.H.L.); gshy2@cam.ac.uk (G.S.H.Y.)

**Keywords:** DEFLATE, compression, Grantham, variation, sequence alignment, nsSNP

## Abstract

Improvements in speed and cost of genome sequencing are resulting in increasing numbers of novel non-synonymous single nucleotide polymorphisms (nsSNPs) in genes known to be associated with disease. The large number of nsSNPs makes laboratory-based classification infeasible and familial co-segregation with disease is not always possible. *In-silico* methods for classification or triage are thus utilised. A popular tool based on multiple-species sequence alignments (MSAs) and work by Grantham, Align-GVGD, has been shown to underestimate deleterious effects, particularly as sequence numbers increase. We utilised the DEFLATE compression algorithm to account for expected variation across a number of species. With the adjusted Grantham measure we derived a means of quantitatively clustering known neutral and deleterious nsSNPs from the same gene; this was then used to assign novel variants to the most appropriate cluster as a means of binary classification. Scaling of clusters allows for inter-gene comparison of variants through a single pathogenicity score. The approach improves upon the classification accuracy of Align-GVGD while correcting for sensitivity to large MSAs. Open-source code and a web server are made available at https://github.com/aschlosberg/CompressGV.

## 1. Introduction

*In-silico* analysis of genetic variants aims to efficiently and accurately predict the presence and penetrance of phenotypic pathogenicity. Focusing on single nucleotide polymorphisms resulting in non-synonymous amino acid (AA) coding (nsSNPs), a number of existing tools provide assessments of predicted classification of variants based on various measures [1–3]. One approach involves multiple-species sequence alignments (MSAs) for analysis of phylogenetic diversity at the same locus as the nsSNP under consideration; although each differs in their analysis, all aforementioned measures implement this means of quantification.

A measure of diversity originally proposed by Grantham [4] was later extended and implemented in Align-GVGD [1] (an explanation of this implementation is outlined below, in Section 2). Greater diversity implies lesser evolutionary hold over the locus and thus a lower probability of deleterious outcome for novel variants. However this approach does not account for the number of species in the MSA and has been shown by Hicks *et al.* [5] to overestimate diversity while, at a critical MSA depth, classifying all nsSNPs as neutral. The aim of our research was to correct for this shortcoming. It is important to note that the specifically curated MSAs provided by the Align-GVGD team are not affected by this scenario; however, these only covered 11 genes at the time of our work.

In the absence of natural selection, random mutations between generations would, with time, render a genetic locus compared between species indistinguishable from a random sequence; the so-called entropy of the sequence increases with time. An analogous effect can be seen when two initially separated gases are allowed to mix; the ordered state of distinct groups of molecules progressively reaches an equilibrium state of indistinguishable mixture.

A rigorous treatment of this phenomenon is found in the second law of thermodynamics, the requirement of the thermodynamic entropy of a closed system to be non-decreasing.

James Clerk Maxwell’s famous thought experiment [6] attempted to violate this law with the aid of a hypothetical demon redirecting molecules by opening and closing a frictionless door. Previously mixed gases would eventually separate much in the same way that homologous genes maintain a level of order; a low entropy distribution of gas molecules is analogous to tight evolutionary control. Natural selection operates as Charles Darwin’s demon; through survival or death, acting to open and close Maxwell’s hypothetical door.

Maxwell’s proposition was later countered by the close similarity between thermodynamic entropy and information entropy [7], a measure of uncertainty in a data source. We recognised that highly controlled, low-information loci would display low Kolmogorov complexity [8] and that an upper bound for such complexity could be determined through data compression. By utilising the compression ratio we could account for expected diversity for MSA depth and correct for the Grantham overestimation. High compressibility should correlate with deleterious nsSNP phenotypes.

## 2. Theoretical Background

Positive evolutionary selection of a non-synonymous AA change has been shown to—on a genome-wide scale—inversely correlate with the respective Grantham score (*GS*), a quantified biochemical difference between individual AAs [4]. Considering a pair of AAs *W* (wild-type) and *V* (variant), the *GS* is the Euclidean distance between chemical properties of *W* and *V*; dimensions are normalised by mean AA distance (considering all 20 AAs) within the same dimension. Grantham’s original definition of *GS* scaled the value by 50.723 such that the average pair-wise distance between AAs would be 100; we ignored this arbitrary adjustment as its linear nature has no effect on our analysis. The *GS* accounts for differences in composition *X_c_*, polarity *X_p_* and molecular volume *X_v_*; with normalisation coefficients *α* = 1.833, *β* = 0.1018, and *γ* = 0.000399:

(1) $$GS=\sqrt{\alpha {\left(W_{c}-V_{c}\right)}^{2}+\beta {\left(W_{p}-V_{p}\right)}^{2}+\gamma {\left(W_{v}-V_{v}\right)}^{2}}$$

An extension of the concept, the Grantham variance (*GV*) is a measure of the diversity amongst a group of AAs [1], in this case a column from the MSA. For the smallest rectangular prism encompassing all residues, the *GV* is the length of the longest diagonal. For a set of AAs *A*:

(2) $$\begin{array}{c} |d|=\underset{X,Y\in A}{\text{max}}{\left(X_{d}-Y_{d}\right)}^{2}\\ GV=\sqrt{\alpha |c|+\beta |p|+\gamma |v|} \end{array}$$

Thus *GS* is equivalent to *GV* for *A* consisting of only 2 AAs.

Align-GVGD includes a metric, *GD*, which is either (i) the shortest Euclidean distance from *V* to the rectangular prism surrounding the set *A*; or (ii) zero if *V* is contained within the said prism. Classification of novel nsSNPs is then achieved by reference to a plane in *GV-GD* space that describes empirically determined probabilities of deleterious outcome. In the limit, the non-decreasing volume of the prism will encompass *V* as MSA depth increases to include more distantly related species, hence the phenomenon described by Hicks *et al*. [5].

### 2.1. Correcting Overestimation of Diversity

Representing the set *A* as an alphabetically sorted ASCII character string *S* we defined *S_c_* as the compressed equivalent when utilising the DEFLATE algorithm [9]. *GV* is adjusted by the ratio of the lengths of the plain and compressed strings with examples provided in Table 1. As *GV* does not necessarily overestimate diversity in a linear manner with respect to Kolmogorov complexity, we incorporated an additional factor *r* to be found empirically.

(3) $$GV^{\prime }=GV\times {\left(\frac{|S|}{|S_{c}|}\right)}^{r}$$

### 2.2. Classification Metric

We combined these measures into a one-dimensional metric for quantitative comparison of nsSNPs. Such a metric would allow for analysis of clustering between sets of known deleterious and neutral variants and thus provide a means for classification of novel ones. An accurate metric should reflect intuition inferred from evolutionary theory and thus account for:

1. Wild-type and variant AA difference; increased *GS* implies greater change and thus a higher metric.
2. Evolutionary constraint of the specific AA position within the protein; increased *GV*′ of an appropriate MSA position reflects greater phylogenetic diversity, implying weaker negative selection and thus a lower metric.
  (a) Complete constraint of a position (*GV*′ = 0) should leave the *GS* component unchanged whilst the limit as *GV*′ approaches infinity should be zero, *i.e.*, absolute evolutionary freedom.

Thus we introduced an empirical, gene-specific constant *k* ≥ 1 and hypothesised that the Grantham Metric (*GM*) is an appropriate pathogenicity metric:

(4) $$GM=GS\times k^{-GV^{\prime }}$$

### 2.3. Empirical Constants

As Grantham’s original work compared the *GS* with the relative swapping frequency across the entire human genome, there was no consideration of gene-specific appropriateness of the coefficients *α*, *β*, and *γ*. We incorporated *c*, *p* and *v* to account for an individual gene’s sensitivity to specific properties.

$$\begin{array}{l} \alpha^{\prime }=c\times \alpha \\ \beta^{\prime }=p\times \beta \\ \gamma^{\prime }=v\times \gamma \\ c,p,v\geq 0 \end{array}$$

### 2.4. Metric Clustering

In order to empirically assign values to *k*, *r*, *c*, *p* and *v*, a measure of fitness was required. As pathogenicity is a binary property, we required a measure of numerical clustering of two sets in one dimension. Increased clustering between the sample sets of deleterious and neutral metrics indicated improved distinction of the two groups by the model.

We utilised the cluster index proposed by Davies and Bouldin [10] who were originally interested in the *similarity* of clusters as against their *distinction* as we are. The index is a function that satisfies two noteworthy criteria: (i) “if the distance between clusters increases while their dispersions remain constant, the [distinction] of the clusters [increases]”; and (ii) “if the distance between clusters remains constant while the dispersions increase, the [distinction] [decreases]” (original text altered to reflect our interest in *distinction*). For sample sets *D* (deleterious) and *N* (neutral) and *x* = *GM*:

(5) $$R(k,r,c,p,v)=\frac{|{\bar{x}}_{D}-{\bar{x}}_{N}|}{s_{D}+s_{N}}$$

where distance is the absolute value of the difference of sample means *χ̄* and the dispersion measure is the sample standard deviation *s*.

### 2.5. Model Training

Maximisation of the clustering index was achieved by computational means. We utilised a Particle Swarm Optimisation (PSO) algorithm [11–13] to achieve greatest clustering.

The swarm *S* contains *n* “particle” vectors, distributed randomly in ℝ^5^ at time *t* = 0, representing candidates for our 5 empirical constants. Each particle *s_i_* ∈ *S* has an associated neighbourhood set *N_i_* ⊂ *S*, constant over *t* and always including itself, *i.e.*, *s_i_* ∈ *N_i_*. Note that the neighbourhood is not necessarily local with respect to the search space, thus allowing for swarm-wide dissemination of information regarding maxima.

Let *S^t^* represent the updated values of the particles at time *t*. Prior to iteration of the optimisation at time *T* the historically optimal candidate vectors are determined both globally (*s_j_* ∈ *S*) and locally (*s_j_* ∈ *N_i_*; 0 *< i < n*):

$$\begin{array}{r} optim_{global}^{T}={\text{argmax}}_{s_{j}^{t}}R(s_{j}^{t})\forall s_{j}^{t}\in S^{t},0\leq t\leq T\\ optim_{i}^{T}={\text{argmax}}_{s_{j}^{t}}R(s_{j}^{t})\forall s_{j}^{t}\in N_{i}^{t},0\leq t\leq T \end{array}$$

Iteration of each vector *s_i_* involves a stochastic traversal of the search space with an attraction *g* and *l* towards $optim_{global}^{t-1}$ and $optim_{i}^{t-1}$ respectively; with the distance to historically optimal points:

$$\begin{array}{c} \Delta_{global}^{t-1}=optim_{global}^{t-1}-s_{i}^{t-1}\\ \Delta_{i}^{t-1}=optim_{i}^{t-1}-s_{i}^{t-1} \end{array}$$

and where $U_{i}^{t}$ are random vectors with elements uniformly distributed [−1*,* 1]:

$$\begin{array}{r} s_{i}^{t}=s_{i}^{t-1}+U_{i}^{t}+g\times \Delta_{global}^{t-1}+l\times \Delta_{i}^{t-1}\\ 0<g,l<1 \end{array}$$

### 2.6. Classification of New Variants

#### 2.6.1. Binary

Binary classification as deleterious or neutral was done by selecting the designation (allocation to set *D* or *N*) that *maximised* the cluster index *R*. Conversely, a *decrease* in *R* would have signified increased *similarity* between the two sets, a sign that the model was separating them less accurately.

It can be shown that the cut-off for designation is:

(6) $$C=\frac{|{\bar{x}}_{D}-{\bar{x}}_{N}|}{2}$$

*i.e.*, for variant AA *V*:

GM>C⇒V∈D

and conversely

GM<C⇒V∈N

#### 2.6.2. Continuous

As the cut-off *C* differs we cannot directly compare pathogenicity metrics between genes. Thus we hypothesised a normalised metric

(7) $$\text{ln }\left(\frac{GM}{C}\right)$$

for modelling with continuous distributions *f_D_*(*x*) and *f_N_*(*x*) for the deleterious and neutral sets respectively.

The models *f_D_*(*x*) and *f_N_*(*x*) can then be used for Bayesian updating of the probability of deleterious classification Pr(*V* ∈ *D*). Given that point probability on a continuous distribution is undefined we consider an infinitesimally narrow region of width *ε* and then limit this to 0. For sufficiently small *ε >* 0, and *M* being the event that $x\leq \text{ln }\left(\frac{GM}{C}\right)<x+ɛ$, first note that the sets *D* and *N* are mutually exclusive and exhaustive, *i.e.*,:

$$\begin{array}{l} \text{Pr}((V\in D)\cap (V\in N))=0\\ \text{Pr}((V\in D)\cup (V\in N))=1 \end{array}$$

$$\begin{array}{l} ∴\;Pr(M)=Pr(M\cap (V\in D))+Pr(M\cap (V\in N))\\ =Pr(M|V\in D)Pr(V\in D)+Pr(M|V\in N)Pr(V\in N) \end{array}$$

(8) $$\begin{array}{l} \text{Pr}(V\in D|M)=\frac{\text{Pr}(M|V\in D)\;\text{Pr}(V\in D)}{\text{Pr}(M)}\\ \approx \frac{ɛf_{D}(x)\;\text{Pr}(V\in D)}{ɛf_{D}(x)\;\text{Pr}(V\in D)+ɛf_{N}(x)\;\text{Pr}(V\in N)}\\ =\frac{f_{D}(x)\;\text{Pr}(V\in D)}{f_{D}(x)\;\text{Pr}(V\in D)+f_{N}(x)\;\text{Pr}(V\in N)}\\ ∴\;\text{Pr }\left(V\in D|x=\text{ln }\left(\frac{GM}{C}\right)\right)=\frac{f_{D}(x)\;\text{Pr}(V\in D)}{f_{D}(x)\;\text{Pr}(V\in D)+f_{N}(x)\;(1-\text{Pr}(V\in D))} \end{array}$$

## 3. Results

The HumVar [3] training data was utilised for testing of the suitability of our algorithm. This is a large dataset of known deleterious and neutral (*MAF >* 1%) variants, grouped by gene. Pre-computed MSAs accompanying the data were utilised for a leave-one-out cross-validation; for each gene we iterated through all known variants, individually excluding them and training a model based on the remaining variants of the same gene. The variant in question was then classified under the binary method and the designation was compared with the known classification.

Only genes with at least 3 variants per set were included. This, after exclusion of the variant being classified, allowed for at least 2 remaining variants in order to calculate a meaningful standard deviation for the clustering metric.

The same variants were classified using the Align-GVGD [1] web interface and the SIFT [2] submitting for alignment script that allows specification of MSA. The HumVar-associated MSAs were used and each variant was classified once with SIFT and multiple times with Align-GVGD with varying MSA depth. Comparison with PolyPhen [3] could not be performed with this dataset as it was used to train the classifier and would thus favourably skew results. SIFT returned a classification of NOT SCORED for 242 (4.29%) variants.

### 3.1. Assessment of Classification

#### 3.1.1. Binary

A total of 5645 variants across 212 genes were tested and a Matthews correlation coefficient (MCC) [14] of 0.38 was achieved at the theoretically optimal cut-off *GM* = *C* with a maximal MCC of 0.41 at $\text{ln }\left(\frac{GM}{C}\right)=-0.97$. Compared with random allocation with equal probability of assignment to each set, this equates to *χ*^2^(1) statistics of 833.23 (*p <* 10^−16^) and 968.49 (*p <* 10^−16^) respectively. This remained unchanged when the values *c*, *p*, and *v* were locked at 1 (*i.e.*, using Grantham’s original dimension scales). Allowing *r* to vary for each classification diminished performance and an optimal constant value of 2.47 was found empirically. As such, all further testing was performed in this manner, leaving *k* as the only free variable.

The nature of the value of *r* reflects the relation between the DEFLATE algorithm and evolutionary history. One could not necessarily expect this to be a non-arbitrary value such as an integer nor any common constant and, additionally, it would change should a different compression algorithm be utilised.

We achieved sensitivity of 64.56% and specificity of 83.42% with the confusion matrix shown in Table 2. Exclusion of the DEFLATE adjustment (*r* = 0) reduces sensitivity to 58.11% and specificity to 83.24%; the greater effect on sensitivity is to be expected as this is, as described below, in keeping with Align-GVGD’s decreasing performance with increasing MSA depth.

#### 3.1.2. Continuous

**Figure 1.**
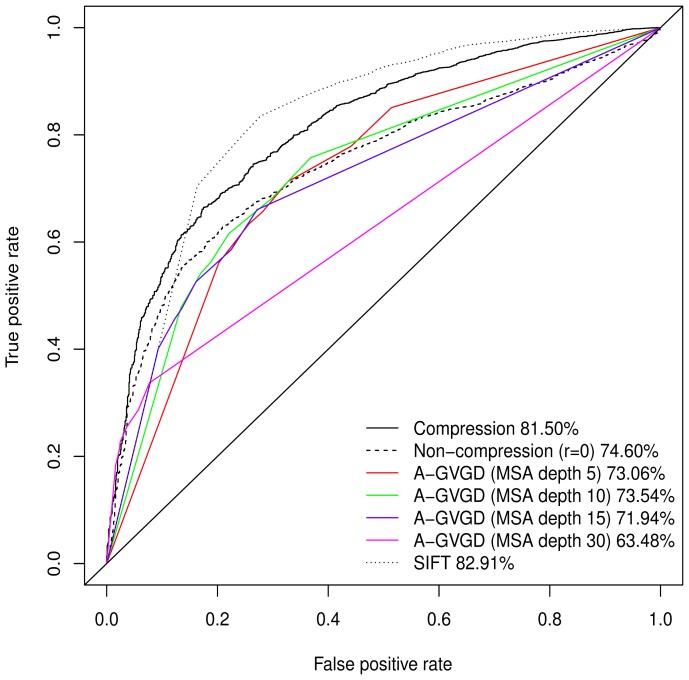
Receiver Operating Characteristic for various classification algorithms. *GM* clustering (full MSA depth) with compression-adjusted *GV* and without compression-adjustment. Align-GVGD with MSA of various depths. Respective AUC displayed as percentage in legend. The progressively deeper MSAs display higher propensities to simultaneously low true and false positive rates, classifying all variants as neutral, in keeping with the findings of Hicks *et al*. [5]. SIFT performed marginally better than compression-adjusted *GM*, but it did not assign a classification to 4.29% of the variants.

Normalised values were used to produce a Receiver Operating Characteristic (ROC) plot with an area under the curve (AUC) of 81.50%. Align-GVGD had a peak AUC of 73.54% (MSA depth of 10), decreasing with greater MSA depth, as originally shown by Hicks *et al*. [5] (Figure 1); the pre-computed MSAs accompanying HumVar are ordered by similarity to the human protein, thus the shallower MSAs used in testing include closely related species rather than the more distantly related ones to which Align-GVGD is sensitive. The peak Align-GVGD performance was poorer than our model even without DEFLATE adjustment, which had an AUC of 74.60%. SIFT classification resulted in an AUC of 82.91%, which was marginally better than our model although, as noted above, not all variants were assigned a classification.

### 3.2. Validation of Clustering Hypothesis

**Figure 2.**
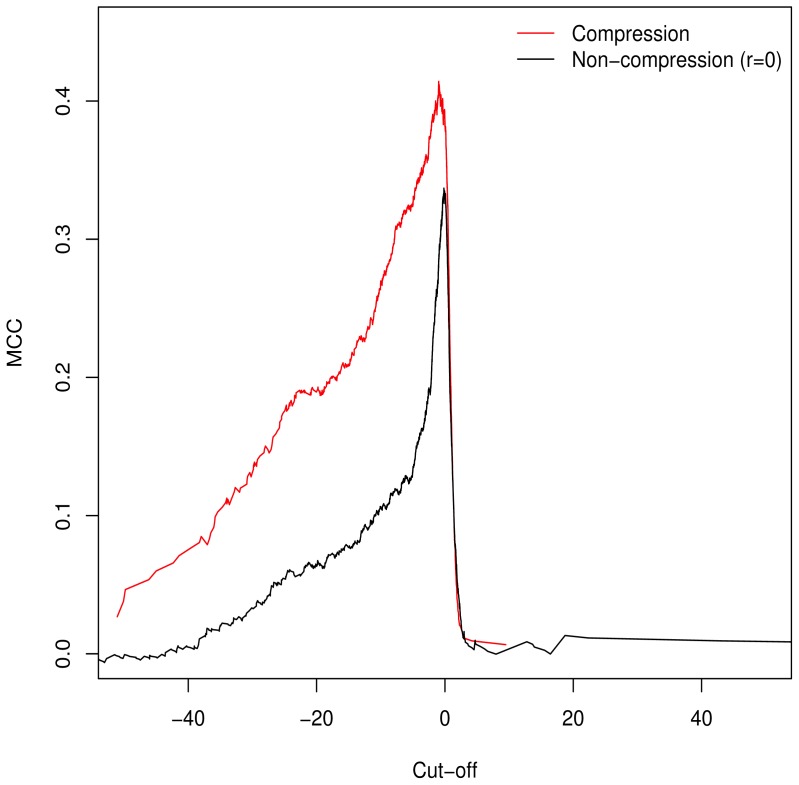
Matthews Correlation Coefficient for all possible cut-off values demonstrates that *GM* = *C* is, in keeping with theory, the empirical optimum. Adjustment of *GV* with compression ratios results in improved performance across the full range of values.

Unlike the AUC, the MCC is defined at a specific cut-off point for binary classification; additionally it is of use in assessing classification accuracy as it controls for differences in the sizes of the positive and negative sets [15,16] (a naive classification model can simply assign all predictions to the larger set, guaranteeing ≥50% accuracy). As such, we used it to determine the empirically optimal cut-off value. The MCC was calculated for the full range of possible cut-off values; Figure 2 demonstrates peaking around $\text{ln }\left(\frac{GM}{C}\right)=0$, which is in keeping with our theorised cut-off of *GM* = *C* as described in Equation (6).

## 4. Discussion

### 4.1. Choice of Binary or Continuous Classification

During the development of our approach, we were questioned as to the need for both methods of classification. Ideally a perfect binary classifier would be produced with 100% certain results. In the absence of such ideal circumstances, we rely on probabilistic measures of certainty such as positive and negative predictive values. Despite this, researchers may require a more nuanced comparison of multiple variants, perhaps in the triage of further laboratory investigations where those with a greater classification metric (despite the same binary classification) may receive preferential attention.

Although progress was made towards determining optimal distributions for *f_D_*(*x*) and *f_N_*(*x*) as required by Equation (8), goodness-of-fit tests showed tail deviation such that we believe that they are currently unsuitable for accurate reflection of probability. Despite this, the normalised value $\text{ln }\left(\frac{GM}{C}\right)$ can still be used for inter-gene comparison of the probability of deleterious outcome; this will provide ordinal relations with magnitudes correlating positively with actual probability. Details of modelling progress are outlined in the Supplementary Information wherein we have demonstrated promising outcomes with a statistically feasible choice of models that result in posterior probability monotonically increasing with respect to $\text{ln }\left(\frac{GM}{C}\right)$.

It has been suggested that in this case only the continuous approach is required; however, the binary method is a logical prerequisite as Equation (7) relies on Equation (6).

### 4.2. Application and Limitations

The scope of evolutionary constraint must be taken into consideration when utilising our model. Genes associated with pathology presenting after reproductive age are intuitively less well suited whilst those associated with congenital abnormalities should be more so. Furthermore, lowered genotypic penetrance will affect results.

Additionally, we recognise that our approach is limited to genes with adequate numbers of known nsSNPs (at least 2 for each of deleterious and neutral). Although our proposed method is not universally applicable, it demonstrates a significant utilisation of additional data where such information is available.

### 4.3. Choice of Classifier

Researchers have many options available for *in-silico* classification of novel nsSNPs. The suitability of each is dependent on individual use cases; as already noted, both our approach and SIFT do not assign classifications to all SNPs whilst Align-GVGD is limited by careful MSA curation. In situations amenable to *GM*-analysis we have demonstrated an objective improvement over Align-GVGD and suggest substitution. Additionally, the Bayesian updating detailed in Section 4.1 is of great benefit in that it allows a simple means of incorporating our methodology into an existing classification pipeline.

## 5. Conclusions

Our results demonstrate that our approach improves performance accuracy when compared with Align-GVGD and that it corrects for sensitivity to large MSAs. Furthermore we have demonstrated that compression ratios of ASCII string representations of AA sets can be used as quantifiers of phylogenetic diversity.

### 5.1. Further Development

Although we have focused our implementation on Grantham-oriented measures, we would like to note the broader applicability of the approach. Incorporation of the compression ratio demonstrates that *GM* can be generalised to use appropriate substitutes or complements to *GS* and *GV* for AA difference and MSA diversity respectively. We welcome suggestions for alternative measures and hope to collaborate on the testing of such alternatives.

## 6. Source Code and Web Server

Compress GV source code (C) is released under the GNU GPL v3; it has been tested under Linux (Ubuntu). A browser interface is available with a link provided in the repository README file. Source code (implementation and browser interface) can be downloaded from https://github.com/aschlosberg/CompressGV.

## Supplementary Information

Although we made progress towards determining optimal distributions for *f_D_*(*x*) and *f_N_*(*x*) as required by Equation (8), goodness-of-fit tests showed tail deviation such that we believe that they are currently unsuitable for accurate reflection of probability. Details of modelling progress are outlined herein.

As we are only interested in non-synonymous AA changes, all *GS* values will be greater than zero and, as such, so too will their associated *GM*. Thus $\text{ln }\left(\frac{GM}{C}\right)$ is, in theory, defined for all variants. In practice (although we never encountered such a situation) rounding can result in *GM* = 0 and such values would need to be excluded prior to modelling.

The Akaike Information Criterion (AIC) [17] is a relative measure of the amount of information [7] lost when modelling data with different distributions. Penalties for the number of model parameters discourage over-fitting of data. The AIC were determined for a series of competing distributions including the skew-t [18] and the generalised hyperbolic [19] family using the R packages *sn* and *ghyp*.

Although absolute values are meaningless, pairwise comparison of model AIC allows for determination of the probability that one truly represents the lower information loss with respect to the other. Deleterious variants are best modelled as an asymmetric generalised hyperbolic distribution whilst the asymmetric normal inverse Gaussian distribution performed best for neutral variants. Of those considered, the next-best performing models are the asymmetric Student-t for both variant sets with probabilities of these secondary distributions in fact being best performing of only 2.46 × 10^−4^ and 2.10 × 10^−26^ respectively. Visual goodness-of-fit analysis and models *vs.* histograms are provided in Figures S1 and S2 respectively. Model parameters and AIC are available in Tables S1 and S2 respectively.

Despite the tail deviations we believe that we have made good progress in defining statistically feasible (*i.e.*, monotonically increasing with respect to $\text{ln }\left(\frac{GM}{C}\right)$) posterior probabilities, defined in Equation (8), as seen in Figure S3.

Figure S1. QQ plots of known variant data (*x*-axis) against samples (*n* = 1,000,000) from lowest-AIC distribution for set as detailed in Table S1. (**a**) Deleterious; (**b**) Neutral.

Figure S2. Empirical and modelled variant distributions based on lowest-AIC distribution for set as detailed in Table S1. (**a**) Deleterious; (**b**) Neutral.

Figure S3. Posterior probability for classification as deleterious based on various prior probabilities. Deleterious (red) and neutral (blue) distributions are shown with posterior probability (black). (**a**) 20% prior; (**b**) 40% prior; (**c**) 60% prior; (**d**) 80% prior.

**Table S1.** Normalised metrics $\text{ln }\left(\frac{GM}{C}\right)$ of known deleterious and neutral variants were modelled with a selection of continuous distributions. Relative loss of Shannon information incurred when modelling with each distribution is determined by the Akaike Information Criteria (AIC). The final column represents the probability, relative to the best performing model, that the model in question incurs minimal information loss.

(a) Known deleterious variants		

Distribution	AIC	Prob.
Generalised Hyperbolic (asym.)	14,128.95	100%
t (asym.)	14,137.26	0.025%
Skew-t	14,202.5	<0.001%
Normal Inverse Gaussian (asym.)	14,203.82	<0.001%
Generalised Hyperbolic	14,983.96	<0.001%
Variance Gamma (asym.)	15,005.94	<0.001%
Normal Inverse Gaussian	15,051.61	<0.001%
t	15,088.24	<0.001%
Hyperbolic (asym.)	15,366.46	<0.001%
Variance Gamma	16,154.94	<0.001%
Hyperbolic	17,097.16	<0.001%
Gauss	22,664.83	<0.001%

(b) Known neutral variants		

**Distribution**	**AIC**	**Prob.**

Normal Inverse Gaussian (asym.)	6014.457	100%
t (asym.)	6073.584	<0.001%
Generalised Hyperbolic (asym.)	6078.023	<0.001%
Skew-t	6099.114	<0.001%
Variance Gamma (asym.)	6266.394	<0.001%
Hyperbolic (asym.)	6296.348	<0.001%
Generalised Hyperbolic	6607.99	<0.001%
Normal Inverse Gaussian	6705.924	<0.001%
Variance Gamma	6803.239	<0.001%
t	6937.972	<0.001%
Hyperbolic	7214.117	<0.001%
Gauss	7894.092	<0.001%

**Table S2.** Maximum likelihood estimates of parameters for models of normalised *GM* displaying lowest AIC. Parameter variables are for the R package *ghyp*.

(a) Deleterious: asymmetric generalised hyperbolic	

**shape (** *λ* **)**	−1.0848428
*χ* **/** *ψ* **shape (** *ᾱ* **)**	0.1518725
**location (** *μ* **)**	0.8148175
**dispersion (** *σ* **)**	1.1393142
**skew (** *γ* **)**	−1.1331262

(b) Neutral: asymmetric normal inverse Gaussian	

**shape (** *λ* **)**	n/a
*χ*/ *ψ* **shape (** *ᾱ* **)**	0.2596805
**location (** *μ* **)**	0.4160513
**dispersion (** *σ* **)**	1.8913547
**skew (** *γ* **)**	−5.3279263

## Figures and Tables

**Table 1.** The compressibility of AA-set strings correlates with evolutionary conservation. *S* represents the ASCII AA-string, and *S_c_* the respective DEFLATE-compressed string.

*S*	|*S_c_*|	$\frac{|S|}{|S_{c}|}$
ACDEFGHIKLMNPQRSTVWY	20	1.00
CCCCCCCCHHRRRRRSSSSS	10	0.50
HHHQQQQQQQRRRRRRRRRR	8	0.40
AAEEEEEEEEEEEEEEEEEE	4	0.20
AAAAAAAAAAAAAAAAAAAA	3	0.15

**Table 2.** Confusion matrix of binary classification as deleterious (D) or neutral (N).

		Actual	

		D	N
**Predicted**	**D**	2924	185
	**N**	1605	931
