# Supplementary figures and images for: DEFLATE Compression Algorithm Corrects for Overestimation of Phylogenetic Diversity by Grantham Approach to Single-Nucleotide Polymorphism Classification
Source: Int J Mol Sci. 2014 May 13;15(5):8491–508. doi: 10.3390/ijms15058491 (PMC4057744; doi:10.3390/ijms15058491)

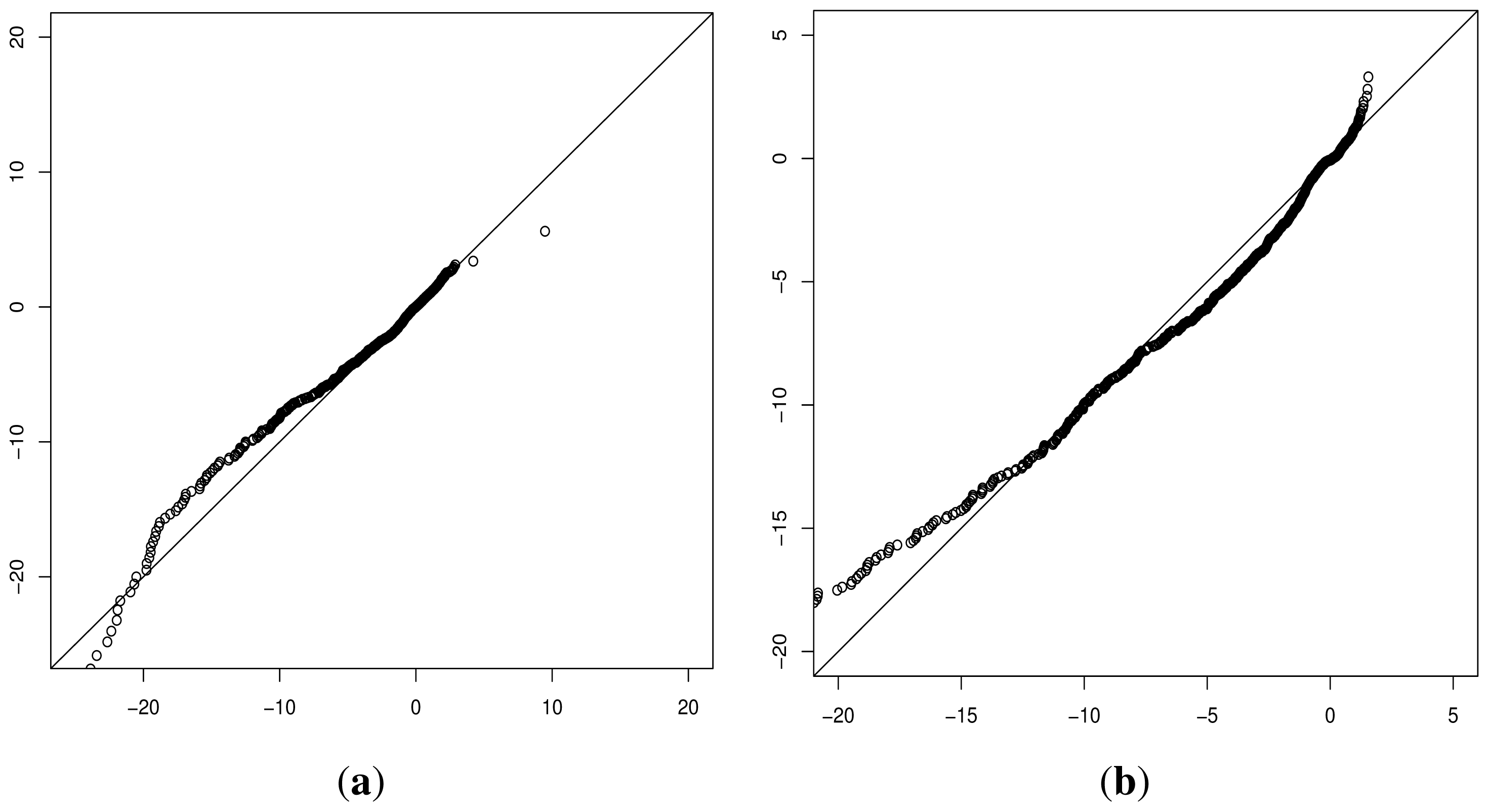

Supplement: Figure S1. — QQ plots of known variant data (x-axis) against samples (n = 1,000,000) from lowest-AIC distribution for set as detailed in Table S1. (a) Deleterious; (b) Neutral. [file ijms-15-08491s1.tif]

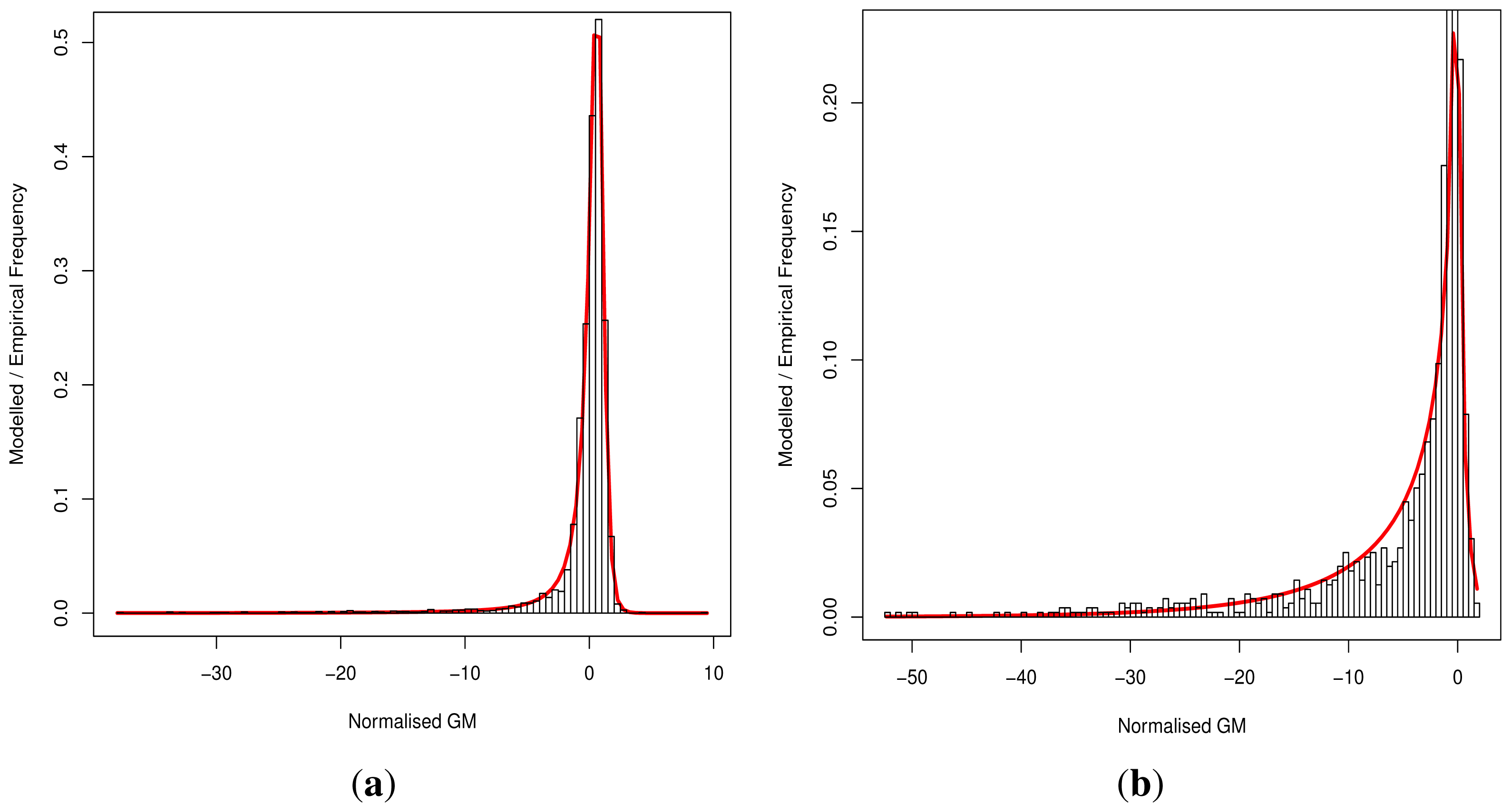

Supplement: Figure S2. — Empirical and modelled variant distributions based on lowest-AIC distribution for set as detailed in Table S1. (a) Deleterious; (b) Neutral. [file ijms-15-08491s2.tif]

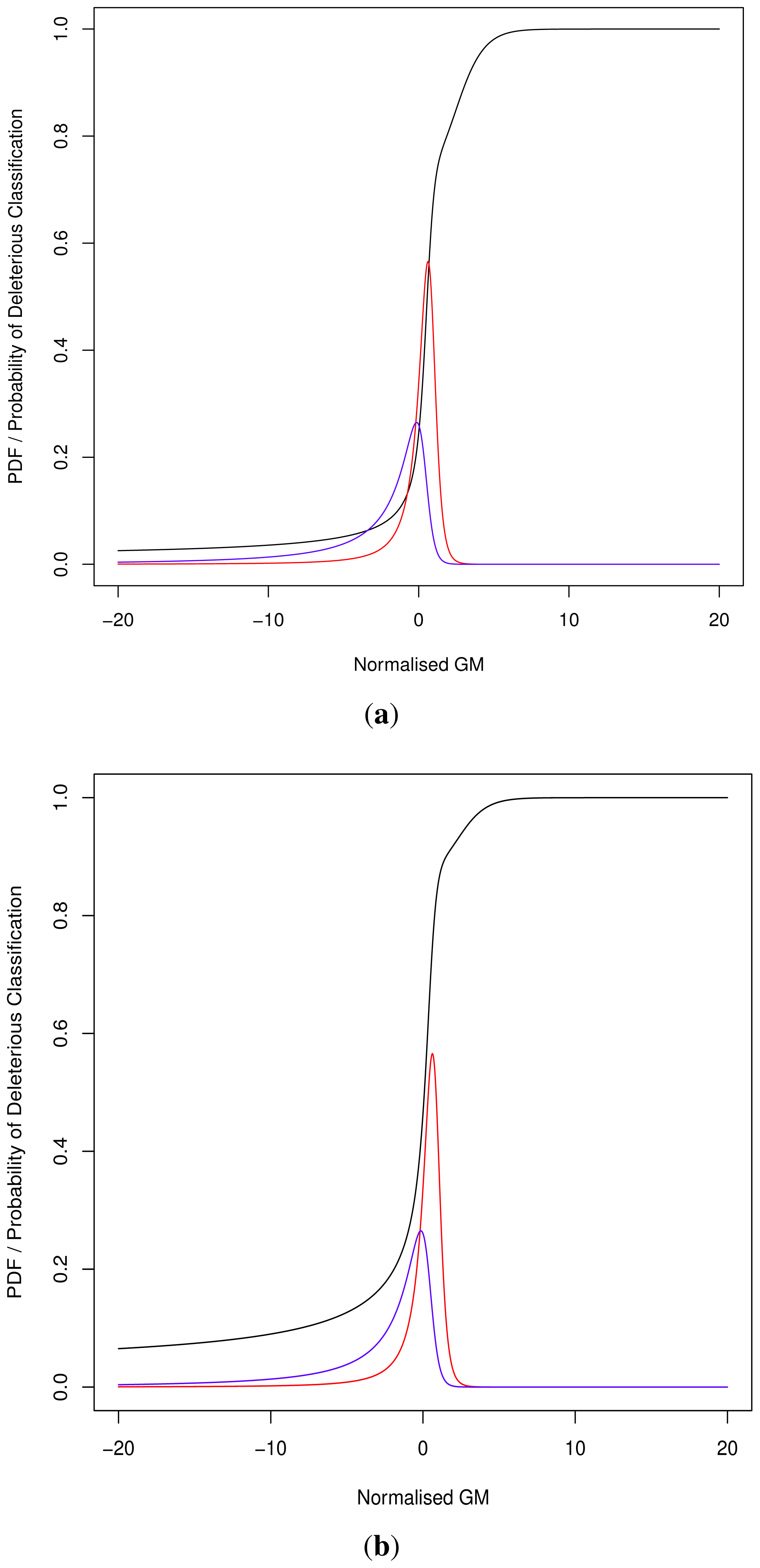

Supplement: Figure S3. — Posterior probability for classification as deleterious based on various prior probabilities. Deleterious (red) and neutral (blue) distributions are shown with posterior probability (black). (a) 20% prior; (b) 40% prior; (c) 60% prior; (d) 80% prior. [file ijms-15-08491s3a.tif]

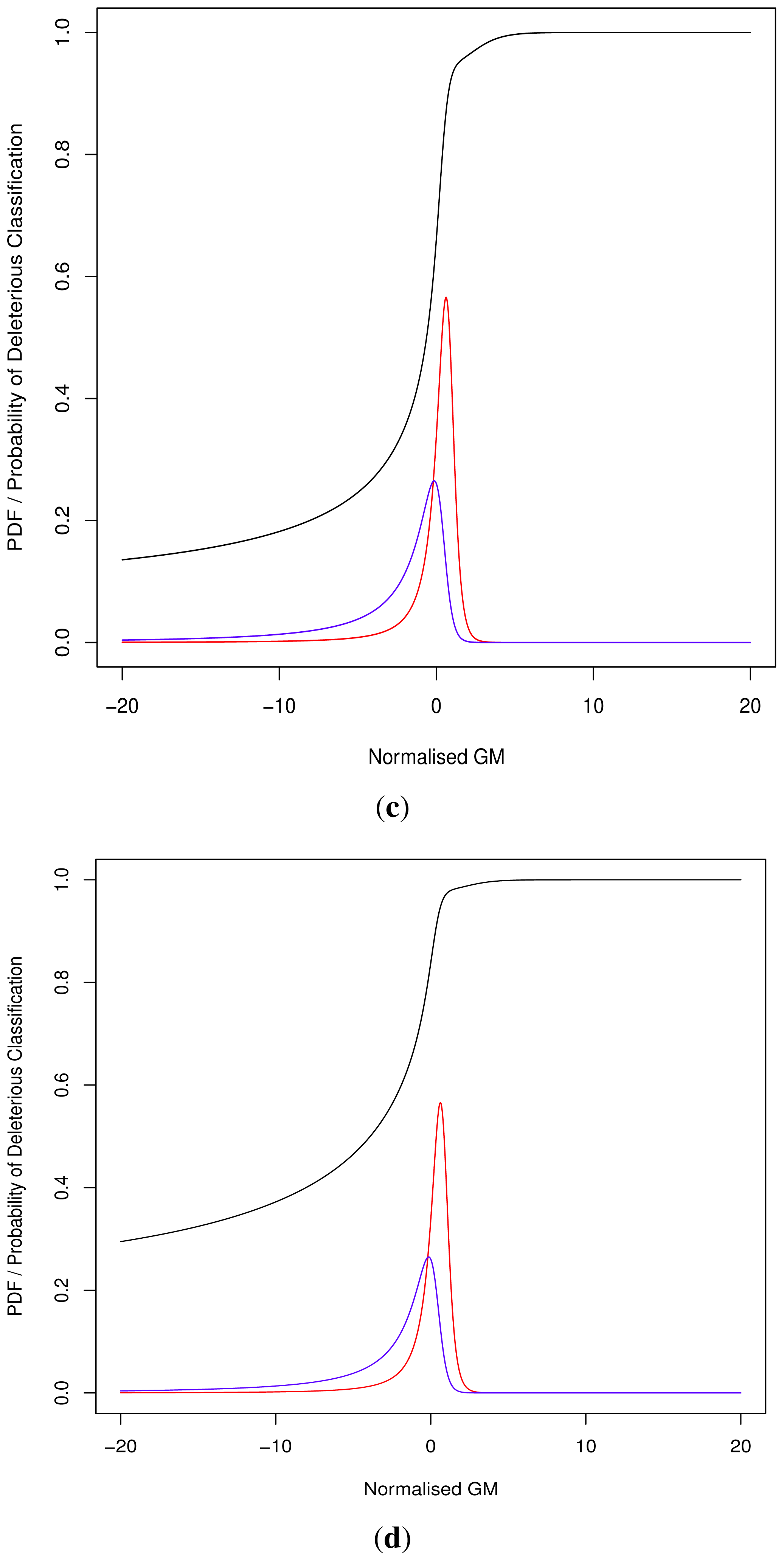

Supplement: Figure S3. — Posterior probability for classification as deleterious based on various prior probabilities. Deleterious (red) and neutral (blue) distributions are shown with posterior probability (black). (a) 20% prior; (b) 40% prior; (c) 60% prior; (d) 80% prior. [file ijms-15-08491s3b.tif]
